# Integrating Clinical and Genomic Analyses of Hippocampal-Prefrontal Circuit Disorder in Depression

**DOI:** 10.3389/fgene.2020.565749

**Published:** 2021-02-05

**Authors:** Naijun Yuan, Kairui Tang, Xiaoli Da, Hua Gan, Liangliang He, Xiaojuan Li, Qingyu Ma, Jiaxu Chen

**Affiliations:** ^1^Formula-Pattern Research Center, School of Traditional Chinese Medicine, Jinan University, Guangzhou, China; ^2^College of Pharmacy, Jinan University, Guangzhou, China; ^3^School of Traditional Chinese Medicine, Beijing University of Chinese Medicine, Beijing, China

**Keywords:** depression, hippocampus-prefrontal circuit, integrative approaches, gene expression, clinical state

## Abstract

Major depressive disorder (MDD) is a prevalent, devastating and recurrent mental disease. Hippocampus (HIP)-prefrontal cortex (PFC) neural circuit abnormalities have been confirmed to exist in MDD; however, the gene-related molecular features of this circuit in the context of depression remain unclear. To clarify this issue, we performed gene set enrichment analysis (GSEA) to comprehensively analyze the genetic characteristics of the two brain regions and used weighted gene correlation network analysis (WGCNA) to determine the main depression-related gene modules in the HIP-PFC network. To clarify the regional differences and consistency for MDD, we also compared the expression patterns and molecular functions of the key modules from the two brain regions. The results showed that candidate modules related to clinical MDD of HIP and PFC, which contained with 363 genes and 225 genes, respectively. Ninety-five differentially expressed genes (DEGs) were identified in the HIP candidate module, and 51 DEGs were identified in the PFC candidate module, with only 11 overlapping DEGs in these two regional modules. Combined with the enrichment results, although there is heterogeneity in the molecular functions in the HIP-PFC network of depression, the regulation of the MAPK cascade, Ras protein signal transduction and Ephrin signaling were significantly enriched in both brain regions, indicating that these biological pathways play important roles in MDD pathogenesis. Additionally, the high coefficient protein–protein interaction (PPI) network was constructed via STRING, and the top-10 coefficient genes were identified as hub genes via the *cytoHubba* algorithm. In summary, the present study reveals the gene expression characteristics of MDD and identifies common and unique molecular features and patterns in the HIP-PFC network. Our results may provide novel clues from the gene function perspective to explain the pathogenic mechanism of depression and to aid drug development. Further research is needed to confirm these findings and to investigate the genetic regulation mechanisms of different neural networks in depression.

## Introduction

Major depressive disorder (MDD) is a highly prevalent and debilitating psychiatric illness that can severely impair quality of life (Belzung et al., 2015). Estimates indicate that depression-related suicide is responsible for the loss of 1 million lives per year (Perlis et al., 2006; Kuo et al., 2015). The physical symptoms of MDD, which are chronic and interfere with daily tasks, behavior, and quality of life, are considered to result from detrimental emotional and cognitive processes.

Since the development of next-generation sequencing, numerous studies have performed transcriptome profiling to explore the mechanisms underlying neural plasticity and brain pathology (Galfalvy et al., 2013; Riglin et al., 2019). Chronic stress can cause wide-ranging changes in gene expression in the human brain, some of which contribute to functional deficits in brain cells (Kajiyama et al., 2010). Many previous expression profiling studies on human samples have identified individual genes as candidate contributors to the depression phenotype, but a comprehensive systematic analysis of the molecular and cellular changes in MDD is still lacking (Iwamoto et al., 2004a; Kroes et al., 2006; Zhang G. et al., 2020). Further analysis of only the representative molecules with the highest differential expression levels will cause the underlying connections among genes to be ignored.

Fortunately, a very well-known method, gene set enrichment analysis (GSEA), can be applied to analyze overall gene expression data among different groups (Subramanian et al., 2005; Reimand et al., 2019). GSEA can be used to identify functional categories or pathways in which genes exhibit coordinated alterations in expression under different kinds of conditions and is not limited to analyses of sets of differentially expressed genes (DEGs). One advantage of GSEA is its ability to highlight and analyze genes with significant phenotypes but relatively minimal changes in expression that may be difficult to detect with classical univariate statistics. Additionally, weighted gene correlation network analysis (WGCNA) is an efficient method that can be used determine the interactions between genes and disease-related phenotypes (Langfelder and Horvath, 2008). This approach involves the analysis and calculation of connectivity weights and topological overlap. The correlation matrix of co-expressed genes and the adjacency function formed by the gene network are defined, and the coefficient of dissimilarity of different nodes is calculated. Then, genes with similar expression profiles are clustered in gene modules. If certain genes always exhibit similar expression changes in the context of a specific physiological process or tissue, the genes are likely functionally related and can be defined as a module. Unlike the clustering criteria of conventional clustering methods (such as geometric distances), the clustering criteria of WGCNA have important biological meaning; thus, the results obtained by WGCNA have high reliability and can be used for numerous further analyses (Rangaraju et al., 2018; Zhang et al., 2019).

Previous autopsy and imaging studies on patients with MDD have indicated the existence of abnormalities in several brain regions, including the prefrontal cortex (PFC), cingulated cortex, hippocampus (HIP), and other brain areas (Li et al., 2015; Mamdani et al., 2015). The HIP-PFC circuit, the critical neural circuit in MDD research, has important functions in cognitive and emotional regulation (Carreno et al., 2016); however, little is known about the gene-related signatures and their correlations in the HIP-PFC neural circuit.

Therefore, we used WGCNA and GSEA with clinical information to explore the phenotype-centric gene networks of patients with MDD. This study used public data sets to ensure a comprehensive analysis, and the included data sets met the following fundamental criteria: (1) all patients had confirmed MDD; (2) complete public transcription data were available; and (3) annotation platform files were available. The available transcriptome datasets on CNS diseases, especially on mental disorders, are limited, and most prior studies focused on a single brain area. Previously, Lanz et al. (2019) collected multiple brain regions from patients with different psychiatric disorders, and systematically evaluated the genes shared between mental diseases. In this analysis, different brain region tissues were collected from the same patient, and all samples are based on the same platform for annotation. Therefore, to explore the transcriptional insights into HPC and PFC in major depression, we performed WGCNA and GSEA of the clinical information in GSE53987, which is the main dataset analyzed in the current study, and other single brain area datasets were incorporated for verification.

## Materials and Methods

### Data Collection and Processing

**FIGURE 1:**
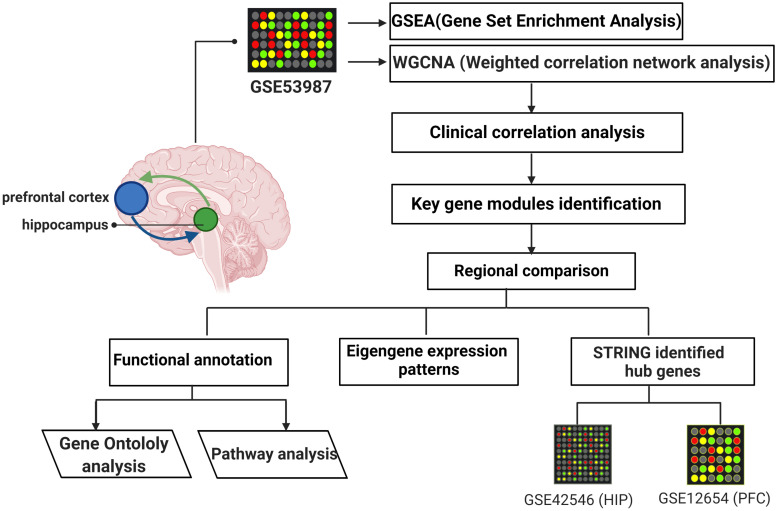
The flow chart of overall study.

Gene microarray datasets (GSE53987, GSE42546, and GSE12654) were retrieved from the Gene Expression Omnibus (GEO) database, and the basic information is listed in Table 1. GSE53987 contains the gene profiles of both the HIP (CTRL/MDD: 18/17) and PFC (CTRL/MDD: 19/17), and no demographic differences were found across the groups (Supplementary Table S1) (Lanz et al., 2019). Under the corresponding platform annotation, 20174 probes can be converted into genes. Then in the verification section, the GSE42546 is a high-throughput sequencing dataset for the HIP, which contains 29 healthy controls and 17 MDD patients with a total of 6297 genes (Kohen et al., 2014). GSE12654 is a gene set for PFC, which includes 15 healthy controls and 11 MDD, and a total of 8622 genes were annotated (Iwamoto et al., 2004b). We used R programming language software to analyze the datasets after converting the corresponding IDs of the probes to the official gene symbols. The subsequent analyses were based on the official gene symbols. Before further analysis, the raw profile data of the CEL files were processed for background correction, and the robust multiarray average (RMA) algorithm of the affy Bioconductor/R oligo package was used for noise reduction and normalized by applying quantile normalization (Gautier et al., 2004). The expression data of each dataset before and after adjustment are displayed in the Supplementary Figures S1, S2. The differential expression analysis of GSE53987 was performed using the R package limma, and the adjusted *P*-value was calculated using Benjamini and Hochberg (BH) correction. Genes with an adjusted *P*-value of <0.05 were statistically significant, log (fold change) > 0 was considered to be upregulated, and <0 was considered to be downregulated compared with the control. For the RNA-seq data (GSE42546), the calcNormFactors function in the edgeR R package was used for correction and normalization (Ritchie et al., 2015). The flow chart of the overall study is shown in Figure 1 (created with BioRender).

**TABLE 1:** Fundamental information of included gene chips.

Data sets	Tissues	Samples (CTRL/MDD)	Platform	Type	Purposes
GSE53987	Hippocampus	18/17	GPL570	Affymetrix U133_Plus2 chips	Analysis
	Prefrontal cortex	19/17			
GSE42546	Hippocampus	29/17	GPL13393	RNA-seq	Validate
GSE12654	Prefrontal cortex	15/11	GPL8300	Affymetrix U95 Version 2	Validate

### Gene Set Enrichment Analysis

GSEA version 3.0 software was used to perform the overall gene analysis on two groups of different samples (control versus depression). To examine whether significant regulatory differences were caused by depression at the functional and pathway levels, background data from the Molecular Signatures Database (MsigDB^1^) were analyzed. The normalized enrichment score (NES) and normalized *P*-value were used to determine statistical significance, as previously described (Subramanian et al., 2005). By calculating the NES, GSEA can resolve differences in gene set size and correlations between gene sets and the expression dataset. The NES value calculation is performed as follow:

$$N\,E\,S=\frac{a\,c\,t\,u\,a\,l\,E\,S}{m\,e\,a\,n\,\left(E\,S\,s\,a\,g\,a\,i\,n\,s\,t\,a\,l\,l\,p\,e\,r\,m\,u\,t\,a\,t\,i\,o\,n\,s\,o\,f\,t\,h\,e\,d\,a\,t\,a\,s\,e\,t\right)}$$

According to the NES calculation method in GSEA, NES can be a positive or negative value. Positive and negative NES values indicate enrichment genes mainly at the top and bottom of the list, respectively. According to GSEA’s instruction and recommendations^2^, the nominal *P*-value estimates the statistical significance of the enrichment scores, thus a conventional 1000 permutation was used in our study to obtain a more accurate *P*-value.

### WGCNA Analysis

To determine the interaction between genes and the disease-related phenotype, an expression matrix was calibrated though the system biology method using the R package WGCNA (Langfelder and Horvath, 2008). This method used gene expression data from two sample groups (the control and MDD groups in this study) to build co-expression pairwise correlation matrices. Because low-expressing or unvarying genes usually means noise, the expression matrix was pre-processed by calculating the variance and screening the genes in the top 50% of the variance. The algorithm assumes that the network is an adjacency matrix *a*_*ij*_, and the co-expression similarity S_*ij*_ is defined as the absolute value of the correlation coefficient between the profiles of nodes i and j based on the default method, S_*ij*_ = | *cor*(*x*_*i*_, *x*_*j*_)|. Hereafter, the pickSoftThreshold function was used to calculate the soft threshold β when constructing each module. Then, by defining the gene co-expression correlation matrices, the algorithm constructs a hierarchical clustering tree and different gene expression modules. For each module, the quantification of module membership (MM) is defined as the correlation between the module eigengene (ME) and the gene expression profile. The modules are defined as having different degrees of clinical relevance based on the calculation of the association between ME and external features. The gene significance (GS) is represented as the correlation (absolute value) between the gene and the clinical traits. For the intramodular analysis of each module, a significant correlation between GS and MM indicates that the genes within that module have consistency with clinical traits. A module was considered important and analyzed further if it correlated with clinical traits and its internal genes significantly correlated with clinical properties. The important modules related to MDD were screened for further analysis.

### Enrichment Analyses for Key Modules

Gene Ontology (GO) enrichment analysis was carried out to determine the key gene modules in the biological process (BP) category. Pathway enrichment analysis was carried out for Kyoto Encyclopedia of Genes and Genomes (KEGG) pathways and Reactome pathways. All genes in the genome were used as the enrichment background. The enrichment analysis was based on the Metascape platform (Saldanha, 2004; Zhou et al., 2019). The *P*-value was calculated for each enriched term (Hochberg and Benjamini, 1990). Terms with *P*-values < 0.05, and enrichment factors (ratios between the observed counts and the counts expected by chance) > 1.0 were clustered based on their membership similarities. More specifically, the *P*-values were calculated based on the cumulative hypergeometric distribution. Kappa scores were used as the similarity metric when performing hierarchical clustering on the enriched terms, and sub-trees with a similarity of >0.3 were considered to form a cluster. The most statistically significant term within a cluster was chosen to represent the cluster.

### Identification and Validation of Hub Genes

The hub genes were identified based on protein interaction evidence from the STRING database (Altaf-Ul-Amin et al., 2006). Evidence for protein interaction of significantly dysregulated genes in key modules were retrieved from the STRING database, which required an interaction score with high confidence (0.7). The Cytoscape plugin *cytoHubba* was used to rank nodes based on the Maxial clique centrality (MCC) topological method, which predicts performance (Chin et al., 2014), and the top-10 genes were selected as hub genes for verification. MCC assumes that the node network is an undirected network; given a node *v*, S(*v*) is the set of the maximal cliques containing *v*, and (| C| −1)! is the product of all positive integers less than | C |. The calculation is as follows:

$$M\,C\,C\,\left(V\right)=\sum_{C\in S\,\left(v\right)}\left(\left|C\right|-1\right)!$$

The separate datasets GSE42546 (HIP) and GSE12654 (PFC) were analyzed to verify gene expression with unpaired two-samples Wilcoxon tests.

## Results

### GSEA for Different Brain Regions in MDD

GSEA was utilized to explore the functions and signaling pathways of gene sets that differed in the MDD groups compared with controls in different brain regions and thus to determine the potential biological significance of these genes sets in MDD. As listed in Supplementary Table S2, the HIP genes in the MDD group were primarily enriched for the GO processes of “dorsal/ventral neural tube patterning,” “negative regulation of protein exit from endoplasmic reticulum” and “regulation of GTP binding,” and the suppressed HIP genes were enriched for the “neuron remodeling,” “serotonin secretion,” and “synapse pruning” processes. The GSEA-based pathway results for the HIP indicated that several critical upstream pathways were suppressed in MDD, such as the “NF-kappa B signaling pathway” and the “TGF-beta signaling pathway”; downstream neuron-related pathways were also inactivated, such as the “GABAergic synapse” and “neuroactive ligand-receptor interaction” pathways (Supplementary Table S2).

For the PFC region, the GSEA results based on GO terms suggested that “stress-induced intrinsic apoptotic signaling pathway,” “negative regulation of fibrinolysis,” and “regulation of neuron projection arborization” were significantly activated in MDD (Supplementary Table S2). Moreover, pathways and GO terms related to synaptic functions and metabolic processes, such as “negative regulation of long-term synaptic potentiation,” “synapse pruning,” and the glutathione and glycerolipid metabolism pathways, were the most inhibited pathways/processes in MDD.

### Key Gene Module Identification

**FIGURE 2:**
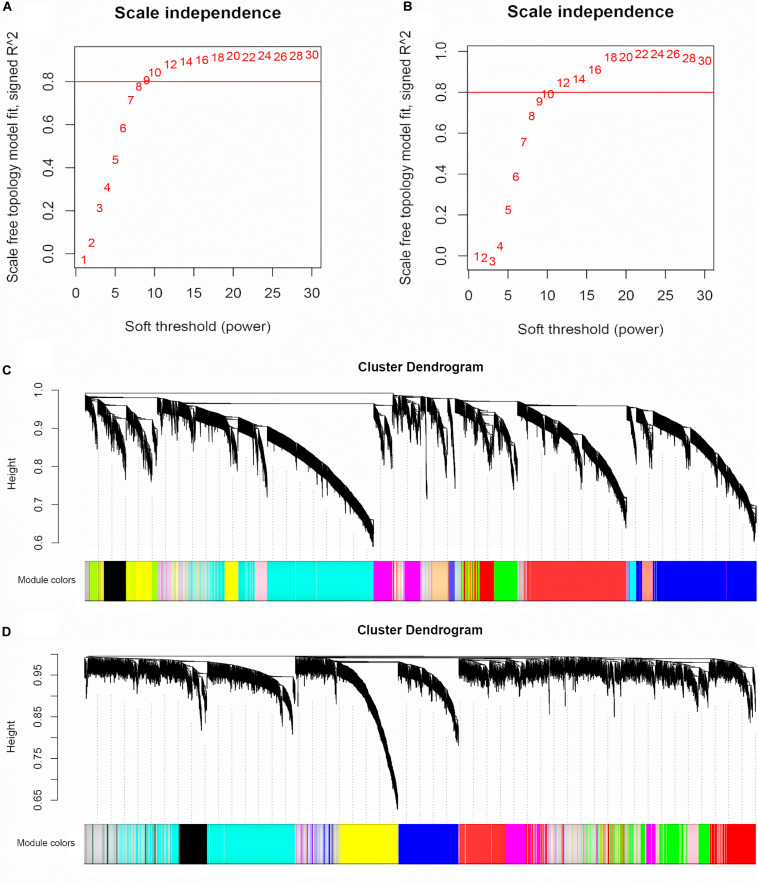
Selecting the appropriate soft threshold and network construction in WGCNA. **(A)** Soft threshold filtering for HIP. **(B)** Soft threshold filtering for PFC. The horizontal axis is Soft threshold (power), and the vertical axis is the evaluation parameter of the scale-free network. The red horizontal line can clearly select the soft threshold when *R*^2^ = 0.8. **(C)** Cluster dendrogram for HIP. **(D)** Cluster dendrogram for PFC. Hierarchical clustering tree showing each module, different colors represent different gene modules.

The input for WGCNA contained all the gene profiles in the GSE53987 dataset; the corresponding clinical characteristics for different brain regions were analyzed separately. The R package WGCNA was applied to classify genes with similar expression patterns into different modules. We first selected a suitable soft threshold β value that met the scale-free conditions (*R*^2^ = 0.9; HIP: 14; PFC: 12; Figures 2A,B). As shown in Figures 2C,D, we used a tree-cutting algorithm to calculate the average linkage clustering, obtain gene co-expression modules, merge similar modules, and gradually build a co-expression network. Ultimately, the gene modules were identified in the dataset for each brain area (HIP: 10; PFC: 12).

**FIGURE 3:**
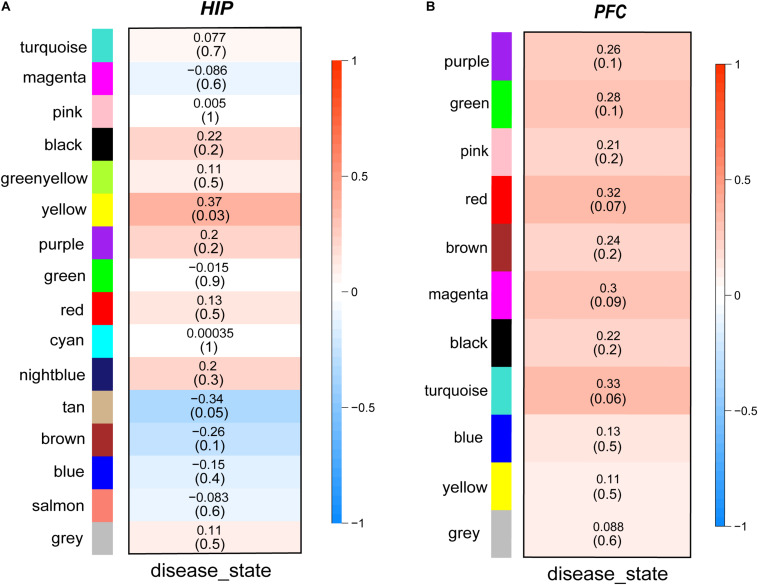
**(A,B)** Module-trait relationships heatmaps. The heatmaps show the clinical relevance of different modules when the diagnosis status is MDD. The intensity of the color represents the strength of the correlation of modules. Red represents positive correlation and blue represents negative correlation. The specific correlation value and *P*-value are displayed in the corresponding module.

**FIGURE 4:**
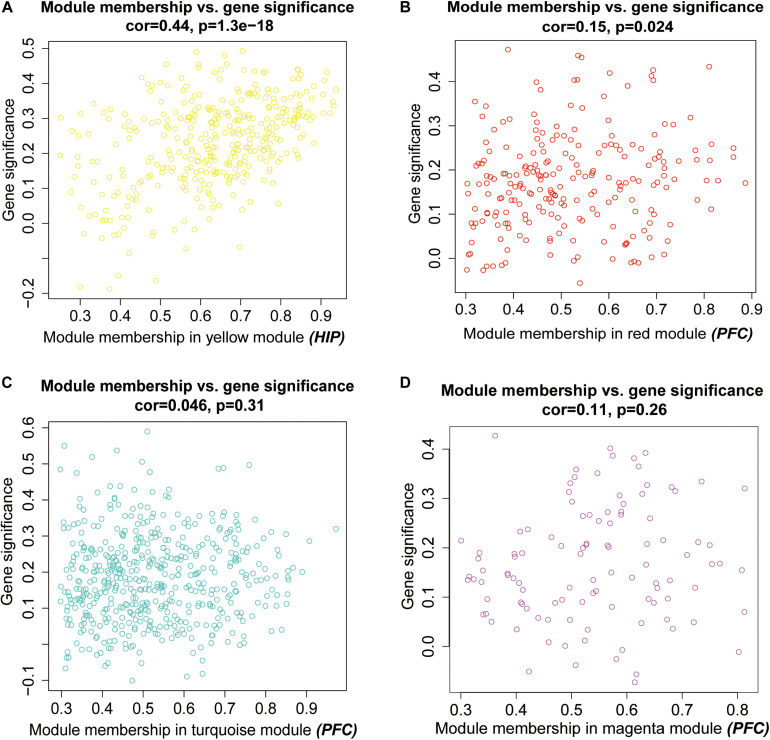
Scatter plots for intramodular analysis. Each dot represents a gene, and different colors represent its module. **(A)** the intramodular analysis for the yellow model in hippocampus, **(B–D)** the intramodular analysis for the modules in prefrontal cortex.

Then, we performed a relevance test to relate each module to clinical diagnostic parameters. The heatmap shown in Figure 3 clearly indicates the clinical relevance of each module with an MDD diagnostic status. The clinical correlation analysis results suggested that the yellow module in the HIP ranked higher than any other module (Figure 3A) and was significantly related to clinical MDD (*r* = 0.37, *P* = 0.03). In the PFC (Figure 3B), the red module (*r* = 0.32, *P* = 0.07), magenta module (*r* = 0.30, *P* = 0.09), and turquoise module (*r* = 0.33, *P* = 0.06) ranked higher than other modules correlated with MDD. We then calculated the relationships between the nodes and modules (Figure 4). The correlation results indicated that the genes within the yellow module in the HIP were significantly correlated (cor = 0.44, *P* = 1.3e–18), and the genes within the red module in the PFC were significantly correlated (cor = 0.15, *P* = 0.024). These modules were consistent with the module analysis results, so they were identified as key modules for subsequent analyses (Figure 4). Additionally, the different modules contained different numbers of genes: the blue module in the HIP contained 363 genes, and the red module in the PFC contained 225 genes. Other genes were not classified into these modules because they lacked clinical relevance or co-expression characteristics; therefore, the modules containing these genes were not further analyzed.

### Regional Comparison of Eigengene Expression via MDD Modules

**FIGURE 5:**
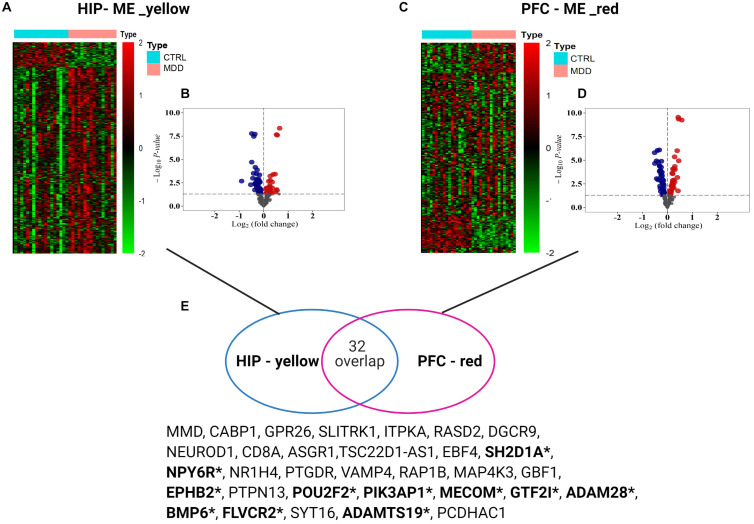
**(A,C)** Heatmaps for key modules. The heatmaps of the modular genes expression levels between groups used the data normalization method of *Z*-score standardization. **(B,D)** Volcano plots for key modules. Gray dots mean genes with no significant difference, red color present up-regulated genes, and blue color dots indicate down-regulated genes **(E)** Venn diagram of gene intersection between two modules, *means the overlapping significant differentially expressed genes.

To analyze the expression patterns of the MDD co-expression modules of the two brain regions, heatmaps were generated (Figures 5A,C). In the yellow module of HIP, the MDD co-expression genes have a different expression pattern than the normal control, with most showing upregulated gene expression; however, in the red module of PFC, the expression patterns of the module genes are not as clear as in HIP, and a wide range of differential expression is shown compared with the control group. To clearly determine the significance compared with the control, we combined the results of the differential analysis of the expression profiles (adjusted *P*-value < 0.05, Figures 5B,D and Supplementary Table S2). Among the 343 genes in the yellow module of HIP, 45 genes were significantly upregulated and 50 genes were significantly downregulated (Figure 5B). Compared with HIP, relatively few DEGs were identified in the PFC red module. Of the 225 genes in the PFC module, 22 genes were significantly upregulated and 29 were significantly downregulated (Figure 5D).

Interestingly, for these two gene sets based on clinical co-expression, few overlapping genes were found (32, Figure 5E). Eleven genes were differentially expressed in both HIP and PFC with similar expression trends. Among these intersecting dysregulated genes, 10 genes were significantly downregulated in HPC and PFC compared to the control group, and BMP6 was significantly upregulated in both brain regions (Supplementary Table S3).

### Regional Comparison of Functions via MDD Modules

**FIGURE 6:**
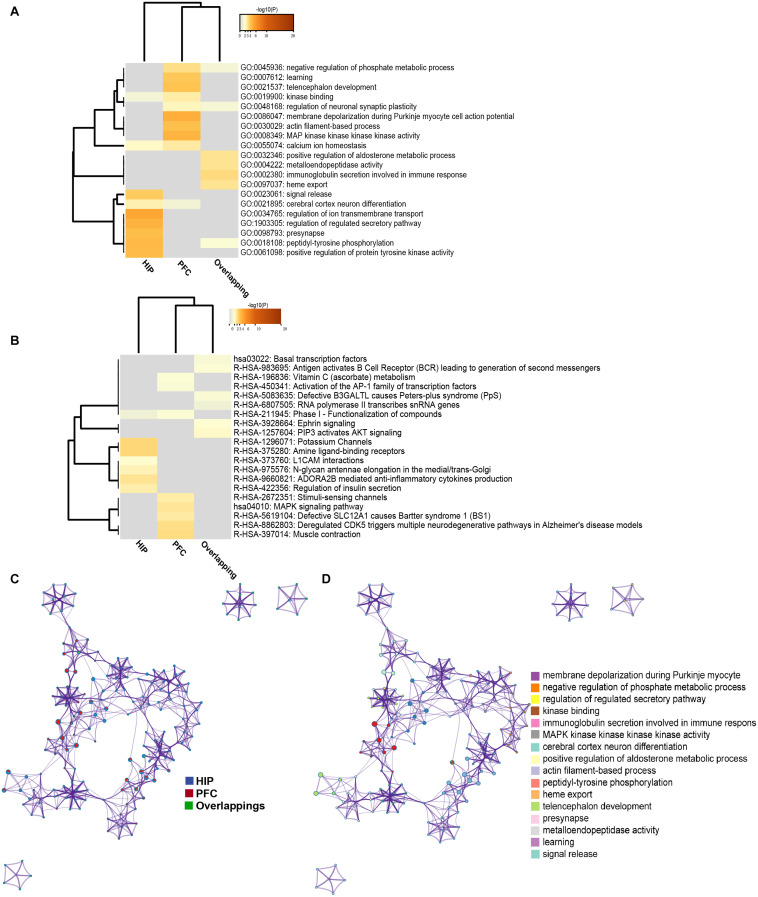
**(A)** GO enrichment heatmap for regional specific DEGs (HIP, PFC) and overlapping DEGs. **(B)** KEGG enrichment heatmap for regional specific DEGs (HIP, PFC) and overlapping DEGs. Gray indicates terms have not been enriched, and the shade of orange represents the degree of enrichment. **(C)** Network of enriched terms represented as pie charts, where pies are color-coded based on the identities of the gene lists. **(D)** Network of enriched terms for HIP and PFC, colored by cluster name, where nodes that share the same cluster ID are typically close to each other.

To analyze the functional differences between the two key co-expression modules, we identified the BP and pathway characteristics with the Metascape database, which can provide GO clusters to more clearly define the enrichment results. The top significant BP enrichment clusters for each brain region are shown in Figure 6A and Table 2. The findings indicate significant differences between the HIP and PFC regions; the depression-related genes of the HIP are most significantly involved in processes associated with neuronal interaction and synaptic structure, such as signal release and cerebral cortex GABAergic interneuron migration, while the depression-related genes of the PFC are significantly involved in processes related to cellular component organization and development, such as telencephalon development, membrane depolarization and MAPKKK activity. The enriched pathways of HIP and PFC have distinguishable attributes (Table 3 and Figure 6B); the HIP yellow module dysregulated genes were significantly enriched in signaling pathways associated with signal transmission between neurons, and synaptic transmission. The top enriched pathways included “Amine ligand-binding receptors,” the downstream signaling pathways of neuronal inflammation and immunity, and “ADORA2B mediated anti-inflammatory cytokines production.” The dysregulated genes in the red module of the PFC participate in the “MAPK signaling pathway” and downstream pathways of molecular metabolism.

**TABLE 2:** GO enrichment for key modules.

GO_ID	Description	*P*-value		
Co-terms		(*HIP*)	(*PFC*)	*(Overlapping)*
GO:0002380	Immunoglobulin secretion involved in immune response			0.0005
GO:0032346	Positive regulation of aldosterone metabolic process			0.0009
GO:0097037	Heme export			0.0009
GO:0004222	Metalloendopeptidase activity			0.0009
GO:0001602	Pancreatic polypeptide receptor activity			0.0018
GO:0043409	Negative regulation of MAPK cascade			0.0031
GO:0036312	Phosphatidylinositol 3-kinase regulatory subunit binding		0.01791	0.0045
GO:0018108	Peptidyl-tyrosine phosphorylation	3.3E-05		0.0116
GO:0046580	Negative regulation of Ras protein signal transduction	0.01252		0.0228
GO:0048168	Regulation of neuronal synaptic plasticity		0.00399	0.0232
GO:0045936	Negative regulation of phosphate metabolic process		0.00067	0.0285
GO:2000474	Regulation of opioid receptor signaling pathway	0.00664	0.00361	
GO:0048167	Regulation of synaptic plasticity	0.00366	0.00482	
GO:0055074	Calcium ion homeostasis	0.00551	0.00174	
GO:0098662	Inorganic cation transmembrane transport	0.001	0.00243	
GO:0021895	Cerebral cortex neuron differentiation	0.00312	0.0442	
GO:0009410	Response to xenobiotic stimulus	0.01562	0.01514	
GO:0031749	D2 dopamine receptor binding	0.01651	0.009	
GO:1905702	Regulation of inhibitory synapse assembly	0.01323	0.0072	
GO:0019900	Kinase binding	0.03684	0.00204	
**Hippocampus**				
GO:0021853	Cerebral cortex GABAergic interneuron migration	0.00016		
GO:0023061	Signal release	0.00016		
GO:0071398	Cellular response to fatty acid	0.00091		
GO:0008422	Beta-glucosidase activity	0.00016		
GO:0098793	Presynapse	6E-05		
GO:0034765	Regulation of ion transmembrane transport	4.6E-06		
GO:0061098	Positive regulation of protein tyrosine kinase activity	3.6E-05		
GO:1903305	Regulation of regulated secretory pathway	1.7E-05		
GO:0019905	Syntaxin binding	0.00209		
GO:0034373	Intermediate-density lipoprotein particle remodeling	0.00332		
**Prefrontal cortex**				
GO:0007612	Learning		0.00013	
GO:0021537	Telencephalon development		0.0001	
GO:0021549	Cerebellum development		0.00091	
GO:0051899	Membrane depolarization		0.0006	
GO:0030029	Actin filament-based process		7.1E-05	
GO:0008349	MAP kinase kinase kinase kinase activity		1.9E-05	
GO:0086047	Membrane depolarization during Purkinje myocyte cell action potential		9.5E-06	
GO:0004597	Peptide-aspartate beta-dioxygenase activity		0.00181	
GO:0052593	Tryptamine:oxygen oxidoreductase (deaminating) activity		0.00361	
GO:0005668	RNA polymerase transcription factor SL1 complex		0.00361	

**TABLE 3:** Pathway enrichment for key modules.

Pathway_ID	Description	*P*-value		
Co-terms		(*HIP*)	(*PFC*)	*(Overlapping)*
R-HSA-1257604	PIP3 activates AKT signaling			0.00603
R-HSA-3928664	Ephrin signaling			0.00855
R-HSA-983695	Antigen activates B Cell Receptor (BCR) leading to generation of second messengers			0.01435
R-HSA-5083635	Defective B3GALTL causes Peters-plus syndrome (PpS)			0.01658
hsa03022	Basal transcription factors			0.02013
R-HSA-6807505	RNA polymerase II transcribes snRNA genes			0.03291
R-HSA-211945	Phase I – Functionalization of compounds	0.04864	0.01575	
**Hippocampus**				
R-HSA-375280	Amine ligand-binding receptors	0.00037		
R-HSA-1296071	Potassium Channels	0.00039		
R-HSA-9660821	ADORA2B mediated anti-inflammatory cytokines production	0.00105		
R-HSA-422356	Regulation of insulin secretion	0.00225		
R-HSA-975576	*N*-glycan antennae elongation in the medial/trans-Golgi	0.00337		
R-HSA-451326	Activation of kainate receptors upon glutamate binding	0.00507		
R-HSA-373760	L1CAM interactions	0.00735		
R-HSA-190374	FGFR1c and Klotho ligand binding and activation	0.00994		
R-HSA-1299503	TWIK related potassium channel (TREK)	0.00994		
R-HSA-8873719	RAB geranylgeranylation	0.01983		
R-HSA-391908	Prostanoid ligand receptors	0.02953		
R-HSA-8984722	Interleukin-35 Signaling	0.03918		
R-HSA-375281	Hormone ligand-binding receptors	0.03918		
R-HSA-1170546	Prolactin receptor signaling	0.04873		
**Prefrontal cortex**				
hsa04010	MAPK signaling pathway		0.00114	
R-HSA-5619104	Defective SLC12A1 causes Bartter syndrome 1 (BS1)		0.00181	
R-HSA-8862803	Deregulated CDK5 triggers multiple neurodegenerative pathways in Alzheimer’s disease models		0.00072	
R-HSA-141405	Inhibition of the proteolytic activity of APC/C required for the onset of anaphase by mitotic spindl		0.03552	
R-HSA-5620916	VxPx cargo-targeting to cilium		0.03726	
R-HSA-450341	Activation of the AP-1 family of transcription factors		0.01791	
R-HSA-196836	Vitamin C (ascorbate) metabolism		0.01436	
R-HSA-1475029	Reversible hydration of carbon dioxide		0.02146	
R-HSA-9008059	Interleukin-37 signaling		0.03726	
R-HSA-2672351	Stimuli-sensing channels		0.0166	
R-HSA-210993	Tie2 Signaling		0.03202	
R-HSA-388844	Receptor-type tyrosine-protein phosphatases		0.03552	
hsa00360	Phenylalanine metabolism		0.03027	

The enriched results revealed identical BPs and pathways both in region-specific DEGs and overlapping DEGs (Tables 2–4 and Figure 6), which indicate common biological changes in the HIP and PFC with regard to coping with MDD, including PIP3-activated AKT signaling, Ephrin signaling and transcription-related downstream signaling pathways. These pathways are involved in the critical BPs of the immune response, and participate in the regulation of the MAPK cascade, neuronal differentiation and synaptic plasticity.

**TABLE 4:** Overlapping DEGs of key modules.

	*PFC*		*HIP*	
Gene	Fold Change	adj.P.Val	Fold Change	adj.P.Val
ADAM28	−1.22718	0.0012	−1.4067	1.44E-05
ADAMTS19	−1.24018	0.0005	−1.1426	0.0129
EPHB2	−1.10922	0.0349	−1.2243	0.0011
FLVCR2	−1.18999	0.0084	−1.1668	0.0120
GTF2I	−1.41651	0.0003	−1.3687	0.0008
MECOM	−1.42665	3.96E-05	−1.4524	0.0039
NPY6R	−1.1682	0.0088	−1.1726	0.0002
PIK3AP1	−1.22638	0.0226	−1.2861	0.0172
POU2F2	−1.19051	0.0007	−1.2575	0.0050
SH2D1A	−1.14261	0.0069	−1.3322	2.80E-08
BMP6	1.31198	2.73E-05	1.27219	0.0024

*adj.P.Val, adjust *P*-value.*

### Hub Gene Selection and Verification

**FIGURE 7:**
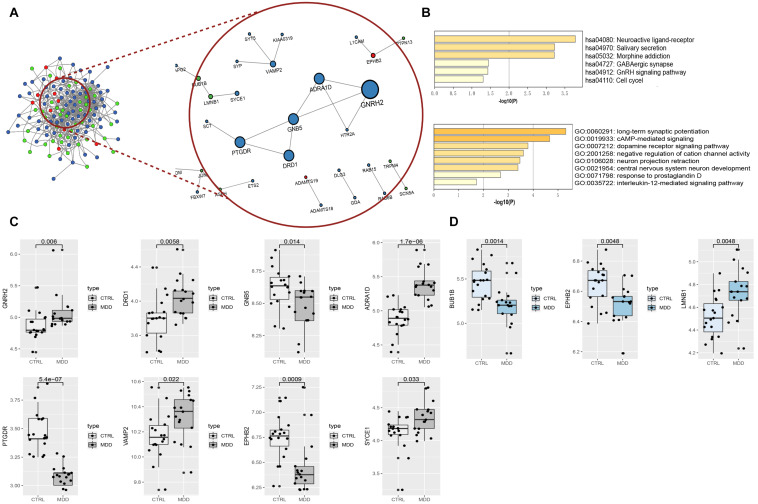
**(A)** PPI network and hub components identified in the HIP and PFC region. Colored by Counts (full connection which according to STRING’s minimum confidence of 0.15); nodes with 0.7 STRING high-confidence nodes only keep in the red circle that shows the high-confidence PPI network and the hub genes identified by *cytoHubba*. The larger the nodes of genes with high coefficients; the red dots represent the common genes of the two brain regions, blue nodes mean HIP genes, and green nodes indicate PFC genes. **(B)** Heatmaps of hub gene enrichment results (pathway and GO biological process). **(C)** Boxplots of hub genes expression in HIP of the analysis set (GSE53987). **(D)** Boxplots of hub genes expression in PFC of the analysis set (GSE53987).

In total, the PPI network annotated by STRING includes 31 points, and the top-10 hub genes were identified though the *cytoHubba* algorithm, among which seven genes in the HIP, two genes in the PFC and EPHB2 in both regions were considered to have strong connections and more primary biological functions than the other genes in the network (Figure 7A and Table 5). The heatmaps were constructed for the related biological functions and signaling pathways for the hub genes (Figure 7B and Table 6). Neuroactive ligand-receptor interaction is the most significantly enriched pathway for these hub genes (ADRA1D, DRD1, PTGDR), and the process of “long-term synaptic potentiation” was regulated by the most hub genes (DRD1, EPHB2, VAMP2, GNB5). In addition, GNRH2, DRD1, ADRA1D, VAMP2, and SYCE1 were significantly upregulated and GNB5 and PTGDR were downregulated in the HIP of MDD patients compared with healthy controls (Figure 7C). BUB1B was significantly downregulated and LMNB1 was significantly upregulated in the PFC of MDD patients. EPHB2, which is involved in the regulation of synaptic enhancement, cation channel activity, neuron projection retraction, central nervous system neuron development and MAPKK activity, was downregulated in both the HIP (*P* = 0.0009) and PFC (*P* = 0.0048) of MDD patients (Figures 7C,D).

**TABLE 5:** Hub node’s information.

Gene	Score	Region-specific	Dysregulated
GNRH2	12	HIP	Up
DRD1	6	HIP	Up
GNB5	6	HIP	Down
ADRA1D	6	HIP	Up
PTGDR	6	HIP	Down
VAMP2	3	HIP	Up
BUB1B	2	PFC	Down
EPHB2	2	both	Down
LMNB1	2	PFC	Up
SYCE1	2	HIP	Up

**TABLE 6:** Enrichment results for hub genes.

Term	Description	*P*-value	Symbols
**Pathway**			
hsa04080	Neuroactive ligand-receptor interaction	0.000164	ADRA1D, DRD1, PTGDR
hsa04970	Salivary secretion	0.000596	ADRA1D, VAMP2
hsa05032	Morphine addiction	0.000609	DRD1, GNB5
hsa04727	GABAergic synapse	0.03554	GNB5
hsa04912	GnRH signaling pathway	0.037128	GNRH2
hsa04110	Cell cycle	0.049748	BUB1B
**Biological progress**			
GO:0060291	Long-term synaptic potentiation	4.83E-06	DRD1, EPHB2, VAMP2, GNB5
GO:0019933	cAMP-mediated signaling	5.05E-05	ADRA1D, DRD1, PTGDR
GO:0007212	Dopamine receptor signaling pathway	0.000162	DRD1, GNB5, VAMP2
GO:2001258	Negative regulation of cation channel activity	0.000248	EPHB2, GNB5
GO:0106028	Neuron projection retraction	0.00041	EPHB2, DRD1
GO:0021954	Central nervous system neuron development	0.000495	DRD1, EPHB2
GO:0071798	Response to prostaglandin D	0.00205	PTGDR, VAMP2
GO:0035722	Interleukin-12-mediated signaling pathway	0.019125	LMNB1
GO:0051301	Cell division	0.024569	BUB1B, SYCE1
GO:0051129	Negative regulation of cellular component organization	0.037388	BUB1B, EPHB2
GO:0051389	Inactivation of MAPKK activity	0.000821	EPHB2

**FIGURE 8:**
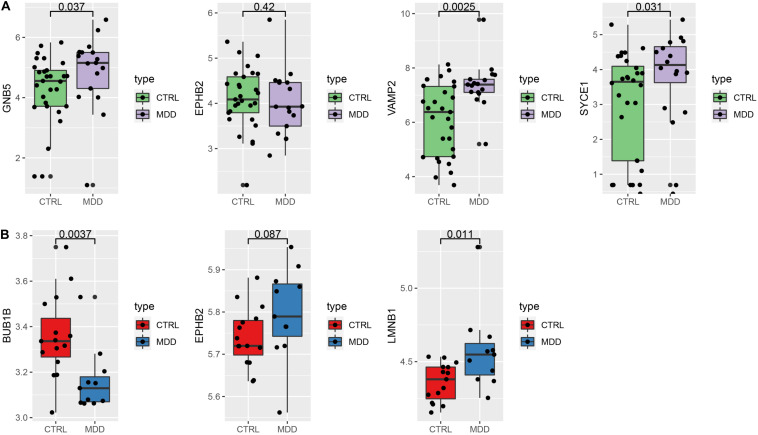
Verify the expression of the hub genes. **(A)** Hub genes expression in the validation set GSE42546 (HIP) **(B)** hub gene expression in the validation set GSE12654 (PFC).

To verify our findings, we used different gene chips to analyze the expression of the hub genes. For the HIP (Figure 8A), 4 hub genes could be annotated in the GSE42546 gene set. The expression of GNB5 (*P* = 0.037), VAMP2 (*P* = 0.0025), and SYCE1 (*P* = 0.031) were significantly upregulated in the HIPs of MDD patients, which is consistent with the results of the analysis set. EPHB2 showed a non-significant decreased expression trend compared to healthy controls in the validation set (*P* = 0.42). Additionally, three hub genes (BUB1B, EPHB2, LMNB1) in the PFC could be annotated in the GSE12654 gene set (Figure 8B). BUB1B was significantly downregulated (*P* = 0.0037), and LMNB1 was significantly upregulated in MDD (*P* = 0.011), but EPHB2 expression did not differ compared with the control (*P* = 0.087).

## Discussion

Building on increasing evidence of the effects of depression on the brain, a previous study revealed that depression is associated with widespread network dysconnectivity rather than aberrant responses of individual brain regions (Li et al., 2018). Both structural and functional abnormalities in different areas have been found in MDD (Park and Friston, 2013; Dai et al., 2019). For example, abnormal functional neural circuitry under chronic stress conditions and decreased volumes of many brain regions have been noted in patients with depression (Korgaonkar et al., 2014). Furthermore, prior studies have revealed the importance of communication from the PFC to the HIP; neural circuitry disinhibition is the main cause of cognitive deficits in depression. Thus, the current study investigated gene signatures in distinct brain areas differentially affected by MDD given that genetic factors have been confirmed to play roles in MDD development (Bast et al., 2017).

First, we used a GSEA method to analyze overall gene data of the HIP and PFC in a holistic manner. The GSEA revealed that the involved genes in the PFC are mainly related to synapse generation and molecular metabolism. For example, the glutathione metabolism pathway is associated with genes that are downregulated in MDD samples. Previous studies reported that glutathione is essential for cellular functions and plays a key role in redox balance *in vivo* mainly by eliminating free radicals to protect cells from oxidative stress (Bi et al., 2020). The current GSEA results for the PFC are consistent with previous clinical data showing that cortical glutathione is dysregulated in MDD. In addition, mounting evidence indicates that glutathione deficits are associated with increased inflammation and oxidative stress in MDD (Lapidus et al., 2014; Lindqvist et al., 2017). The GSEA results for the HIP showed that genes related to neuronal plasticity and serotonin secretion were downregulated and that genes related to the NF-κB signaling pathway and the TGF-β signaling pathway were also dysregulated in MDD samples. Several lines of evidence indicate that TGF-β pathway signaling is necessary for dopaminergic neuron development and function (Tesseur et al., 2017), and TGF-β1 is correlated with pathology in late-onset Alzheimer’s disease and with MDD susceptibility (Zhang K. et al., 2020). The overall results of GSEA indicate that in the context of MDD, the transcriptomic changes in the PFC lead to the abnormal regulation of ion metabolism, while the transcriptomic changes in the HIP lead to abnormalities in the immune and neurotransmitter systems.

To identify specific genes closely related to the progression of depression, we performed WGCNA of these brain areas. Among the genes in the co-expression module of HIP and PFC under major depression conditions, more differences and less gene expression pattern overlap were found, and the HIP yellow module showed more DEGs than the PFC red module. Few overlapping genes were found between regions, indicating decreased expression consistency of HIP and PFC in MDD. With regard to the connections of functions, the two regional co-expression modules also showed heterogeneity. The results point to specific signaling pathways and BP clusters in the PFC and/or HIP. Both brain regions are enriched in the BP of the regulation of neuronal synaptic plasticity; however, the genes in the HIP are still more enriched in processes related to ion transport and signal release, while the depression-related genes in the PFC are significantly enriched in processes related to membrane depolarization and brain tissue development. The enhancement of synaptic potency and the plasticity of neural circuits are considered the primary cellular mechanism of learning and memory. Notably, the PFC has been related to cognitive and executive functions, and PFC dysfunction is indeed a pathological mechanism of depression. In addition, in primates, the HIP is located near the center of the brain and is one of the main limbic brain regions that stores memories and regulates cortisol production; however, its most important function is in adult neurogenesis. Different BPs and signaling pathways are affected in these brain regions in the context of depression due to the physiological distinction between the HIP and PFC. Previous constructive research on schizophrenia suggested that there are few overlapping genes and BPs in the HIP and PFC, which means that the regional consistency in schizophrenia is reduced (Collado-Torres et al., 2019). Our results are consistent with previous studies and provide further molecular biological evidence of the decreased regional connectivity in the depressive state.

Notably, MDD-related genes in both regions were found to be involved in Ephrin signaling, possibly due to the structural changes in neuronal connections in neurological diseases, from synaptic changes to the loss or reconnection of entire axon bundles. Ephrin ligands and their Eph receptors are membrane-bound molecules that mediate the axon guidance through cell-cell contacts, and play a vital role in the guidance of neuronal growth cones, synaptic plasticity, and neuron migration (Torii et al., 2009; Klein and Kania, 2014). Previous studies demonstrated that Ephrin type-B receptor 2 (EPHB2) in the hypothalamus was significantly increased and interacted with the accumulated NMDAR subunit GluN2A in LPS-induced depressive phenotype mice (Wu et al., 2019), and the ratios of p-EphA4/EphA4 and p-ephexin1/ephexin1 in the PFC and HIP were increased in the social frustration depression mouse model (Zhang et al., 2017). In addition, the “MAPK signaling pathway” was significantly enriched in PFC, and “negative regulation of MAPK cascade” and “negative regulation of Ras protein signal transduction” were significantly enriched in the HIP-PFC network. These pathways and biological progresses are involved in the classic brain-derived neurotrophic factor (BDNF)-MAPK pathway. Stress factors alter BDNF activity and influence the BDNF-Ras-MAPK pathway, impairing neuronal cell survival and neuroplasticity, thereby resulting in depression symptoms (Easton et al., 2006). Previous studies have demonstrated that the Ras/MAPK pathway can regulate synaptic plasticity and affect the expression of hippocampal neuronal plasticity-related proteins in mice with chronic depression-like symptoms induced by morphine withdrawal (Zhu et al., 2002; Jia et al., 2013). Overactivation of the Ras/MAPK pathway impacts memory and learning behaviors in rats. Although preceding studies have reported the potential roles of MAPK signaling in depression (Aguilar-Valles et al., 2018), the downstream cascade targets of the MAPK pathway and the roles of their interactions in functional neural circuits in the regulation of depression are still unclear.

The top-10 genes were selected as hub genes from among the DEGs based on the STRING annotation and the *cytoHubba* plugin. The selected genes are involved in BPs such as long-term synaptic potentiation (LTP) and neuron projection retraction, which are representative BPs in the mechanism of depression. LTP and long-term inhibition (LTD) are two opposite forms of synaptic plasticity that contribute to fine-tuning neural connections to store information in the brain. Neural circuit information is disrupted during depression, resulting in learning and memory deficits associated with impaired synaptic plasticity (Adhikari et al., 2010; Padilla-Coreano et al., 2016; Bast et al., 2017). In one study, Zheng and Zhang (2015) clarified the potential causes of synaptic plasticity deficits in the HIP-PFC network during depression and recorded local field potentials (LFPs) in two brain regions of rats under normal and chronic unpredictable stress (CUS). The authors found that impaired synaptic plasticity in the ventral CA1 (vCA1)–medial PFC (mPFC) pathway was reflected in weakened theta coupling and theta-gamma cross-frequency coupling of LFPs in the depressed state. EPHB2 was identified as a hub gene that was significantly downregulated in both the HIP and PFC of depressed patients (analysis set). Even though it did not show significant differential expression in the validation set, many previous studies reported that the receptor tyrosine kinase EphB2 is inactivated in neuropsychiatric disorders including depression and memory disorders. Moreover, EPHB2-knockdown mice exhibit depression-like behaviors and spatial memory deficiency compared with wild-type mice (Zhen et al., 2018). In mice affected by chronic social stress, the expression level of EphB2 and its downstream molecules in mPFC are reduced, and the specific cleavage of the EphB2 receptor can increase sensitivity to stress and induce depression-like behavior (Bouzioukh et al., 2007). The synapses in the HIP of EphB2 mutant mice had normal morphology, but synaptic plasticity was defective and the LTP was attenuated in EphB2(−/−) hippocampal slices (Grunwald et al., 2001).

According to previous reports, HIP-PFC functional circuit interactions mainly occur in the theta frequency range and reflect the processes of working memory. In addition, the active processes in the two brain regions are associated with enhanced oscillatory activities (Spellman et al., 2015); thus, impaired functional neural circuits affect cognitive and memory processes in depression. In addition to discovering only a few overlapping molecular pathways in the HIP-PFC circuit, indicating weak regional coherence, we found that the overlapping pathways may be regularized responses between the HIP-PFC neural circuits that underlie the critical mechanism involved in the pathogenesis of MDD. Therefore, based on the identified gene characteristics of the two brain regions, further research could be performed on the molecular mechanism of this abnormal neural circuit in depression, particularly with regard to the overlapping pathways and several BPs that are closely associated with the HIP-PFC circuit.

The current study has certain limitations. First, most of the current evidence suggests that several brain regions are involved in MDD, including the PFC, HIP, amygdala, thalamus, and STR, and both structural and functional abnormalities in these areas have been found to occur in depression. There are also abnormalities in other neural circuits, such as the basal ganglia-thalamus-cortex circuit, that could be involved in the development of depression; however, we only analyzed the HIP-PFC neural network in this paper, given the few human disease samples and difficulty in analyzing each neural network or neural circuit extensively. WGCNA analysis associates clustered genes with a variety of external clinical information, and the features of clinical depression include the occurrence of anxiety, cognitive symptoms, and sleep phase disorder. This study was conducted based on the current limited clinical information, which may lead to the potential external clinical features of other gene modules not being discovered. This study analyzed the two brain regions of MDD based on the gene expression level, therefore, we verified the DEGs from key modules, however, the analysis and verification of the connection between gene expression and function requires further exploration. Last, we used other gene datasets for verification, but several of the hub genes were not expressed or had no expression differences between the groups. The different annotation platforms and sample sizes might have caused this discrepancy.

Above all, the present study shows the gene expression characteristics of MDD and reveals common and unique molecular features and patterns in the HIP-PFC network. Our results may provide novel clues from the gene function perspective to explain the pathogenic mechanism of MDD and to aid in the development of drug interventions for depression. Further research is needed to confirm our findings and to investigate the mechanisms of gene regulation of different neural networks in depression.

## Data Availability Statement

The original contributions presented in the study are included in the article/Supplementary Material, further inquiries can be directed to the corresponding author/s.

## Author Contributions

NY, QM, and JC conceived of and designed the study. NY, KT, LH, XL, XD, and HG collected and processed the data. NY and KT wrote the manuscript. All the authors contributed to manuscript revision and read and approved the final submitted version. QM and JC take primary responsibility for communication with the journal and editorial office during the submission process, throughout peer review and during publication.

## Conflict of Interest

The authors declare that the research was conducted in the absence of any commercial or financial relationships that could be construed as a potential conflict of interest.

## Footnotes

1 http://software.broadinstitute.org/gsea/msigdb

2 https://www.gsea-msigdb.org/gsea/doc/GSEAUserGuideFrame.html
