# Supplementary material for: Integrating Clinical and Genomic Analyses of Hippocampal-Prefrontal Circuit Disorder in Depression
Source: Front Genet. 2021 Feb 5;11:565749. doi: 10.3389/fgene.2020.565749 (PMC7893101; doi:10.3389/fgene.2020.565749)
Supplement: Supplementary file 1 [file Data_Sheet_1.docx]

**Supplementary materials**

**List**

**Table S1.** The clinical information of GSE53987

**Table S2.** Top 10 GSEA results of HIP and PFC

**Table S3.** Differentially expressed genes of key modules

**Figure S1.** Box plots for the expression data in GSE53987. (A) Data before and after RMA normalization for HIP; (B) Data before and after RMA normalization for PFC

**Table S1. The clinical information of GSE53987**

|  | | | PFC (n=36) | |  | HIP (n=35) | | |
| --- | --- | --- | --- | --- | --- | --- | --- | --- |
|  |  | | CTRL (n= 19) | MDD (n= 17) | ***P*** | CTRL (n= 18) | MDD (n=17) | ***P*** |
| **Age** | | |  |  | 0.43 |  |  | 0.42 |
|  |  | | 48.05±10.65 | 45.18±10.71 |  | 48.17±10.95 | 45.18±10.71 | |
| **Gender** | | |  |  | 0.99 |  |  | 0.87 |
| **Male** | | 10 | | 9 |  | 9 | 9 |  |
| **Female** | | | 9 | 8 |  | 9 | 8 |  |

**Table S2. Top 10 GSEA results of HIP and PFC**

| **ID** | **Description** | **setSize** | **NES** | **qvalues** |
| --- | --- | --- | --- | --- |
| **Pathway-Hippocampus** | |  |  |  |
| hsa04060 | Cytokine-cytokine receptor interaction | 275 | -1.451 | 0.0398 |
| hsa04080 | Neuroactive ligand-receptor interaction | 325 | -1.788 | 0.0204 |
| hsa04514 | Cell adhesion molecules (CAMs) | 134 | -1.67 | 0.0204 |
| hsa04350 | TGF-beta signaling pathway | 88 | -1.561 | 0.0297 |
| hsa04727 | GABAergic synapse | 87 | -1.563 | 0.0402 |
| hsa04659 | Th17 cell differentiation | 102 | -1.624 | 0.0297 |
| hsa04064 | NF-kappa B signaling pathway | 97 | -1.677 | 0.0297 |
| hsa04612 | Antigen processing and presentation | 65 | -1.991 | 0.0204 |
| hsa04672 | Intestinal immune network for IgA production | 43 | -1.995 | 0.0204 |
| hsa05033 | Nicotine addiction | 37 | -1.984 | 0.0204 |
| **Pathway-Prefrontal cortex** | | |  |  |
| hsa04740 | Olfactory transduction | 118 | 1.671 | 0.0345 |
| hsa04145 | Phagosome | 138 | -1.928 | 0.0329 |
| hsa00561 | Glycerolipid metabolism | 53 | -1.763 | 0.0329 |
| hsa00480 | Glutathione metabolism | 51 | -1.872 | 0.0329 |
| hsa05320 | Autoimmune thyroid disease | 46 | -1.851 | 0.0329 |
| **GO-Hippocampus** | |  |  |  |
| GO:0036507 | protein demannosylation | 18 | 1.891 | 0.0021 |
| GO:0006361 | transcription initiation from RNA polymerase I promoter | 34 | 1.736 | 0.0107 |
| GO:0021904 | dorsal/ventral neural tube patterning | 23 | 1.638 | 0.0168 |
| GO:0070862 | negative regulation of protein exit from endoplasmic reticulum | 10 | 1.616 | 0.0223 |
| GO:1904424 | regulation of GTP binding | 11 | 1.533 | 0.0451 |
| GO:0070091 | glucagon secretion | 10 | -1.748 | 0.0059 |
| GO:0035589 | G protein-coupled purinergic nucleotide receptor signaling pathway | 13 | -1.926 | 0.0039 |
| GO:0034134 | toll-like receptor 2 signaling pathway | 14 | -1.945 | 0.0039 |
| GO:0016322 | neuron remodeling | 12 | -2.004 | 0.002 |
| GO:0098883 | synapse pruning | 11 | -2.332 | 0.0019 |
| **GO-Prefrontal cortex** | |  |  |  |
| GO:0051918 | negative regulation of fibrinolysis | 10 | 1.72 | 0.0099 |
| GO:0150011 | regulation of neuron projection arborization | 10 | 1.637 | 0.0178 |
| GO:1902237 | positive regulation of endoplasmic reticulum stress-induced intrinsic apoptotic signaling pathway | 10 | 1.619 | 0.0198 |
| GO:0001963 | synaptic transmission dopaminergic | 24 | -1.598 | 0.0221 |
| GO:1901626 | regulation of postsynaptic membrane organization | 13 | -1.676 | 0.0249 |
| GO:1900272 | negative regulation of long-term synaptic potentiation | 12 | -1.704 | 0.0165 |
| GO:0001973 | adenosine receptor signaling pathway | 11 | -1.752 | 0.0101 |
| GO:0098883 | synapse pruning | 11 | -1.895 | 0.004 |
| GO:0098712 | L-glutamate import across plasma membrane | 13 | -1.988 | 0.0021 |
| GO:1901687 | glutathione derivative biosynthetic process | 20 | -1.99 | 0.004 |

NES: normalized enrichment score

**Table S3. Differentially expressed genes of key modules**

| **genes** | **Fold Change** | **adj.P.Val** |
| --- | --- | --- |
| *HIP* | | |
| ADAMTS18 | -1.884769666 | 0.00174948 |
| MECOM | -1.452437779 | 0.00391209 |
| NR1H4 | -1.43573263 | 1.42E-09 |
| ADAM28 | -1.406651917 | 1.44E-05 |
| GTF2I | -1.368680499 | 0.00080121 |
| WDFY4 | -1.332756103 | 0.00027912 |
| SH2D1A | -1.332244121 | 2.80E-08 |
| PCDHAC1 | -1.286922724 | 2.56E-05 |
| PIK3AP1 | -1.286074151 | 0.01723767 |
| PTGDR | -1.284866869 | 9.59E-09 |
| TSGA10 | -1.281166882 | 0.00348443 |
| POU2F2 | -1.257507879 | 0.0050318 |
| NNAT | -1.25389295 | 0.00328226 |
| SYT16 | -1.238928941 | 0.00243468 |
| TUNAR | -1.232767107 | 0.00413478 |
| GPR22 | -1.224857714 | 0.00035005 |
| EPHB2 | -1.224291462 | 0.00114231 |
| RFPL3 | -1.220773373 | 0.01017344 |
| C2orf80 | -1.212112162 | 0.00214444 |
| LCT | -1.209225266 | 9.25E-05 |
| ETS2 | -1.206122682 | 0.0032525 |
| PGBD1 | -1.19705696 | 0.00239244 |
| RPRML | -1.191257246 | 0.00539717 |
| LOC101928335 | -1.188858481 | 0.01254511 |
| PSG11 | -1.183199639 | 0.00127214 |
| RELL2 | -1.17371497 | 0.0022766 |
| NPY6R | -1.172589827 | 0.00018573 |
| FLVCR2 | -1.166828059 | 0.01198269 |
| SUGT1P3 | -1.1596506 | 0.03984712 |
| SLC45A1 | -1.159469235 | 0.0011193 |
| KCNA6 | -1.157398044 | 0.01867446 |
| RAB15 | -1.143381993 | 0.00337628 |
| FBXW7 | -1.142743832 | 0.01283084 |
| ADAMTS19 | -1.142642221 | 0.01293311 |
| KCNK4 | -1.140907619 | 0.00927078 |
| TSPAN13 | -1.13271574 | 0.00225705 |
| JAKMIP1 | -1.127752589 | 0.00041952 |
| THEMIS2 | -1.126053389 | 0.04731115 |
| FEZF2 | -1.125461067 | 0.02077839 |
| KCNQ2 | -1.124001036 | 0.02395546 |
| SKAP1 | -1.117235794 | 0.0365259 |
| LHFPL4 | -1.11209866 | 0.02661356 |
| FXYD7 | -1.109641969 | 0.0246569 |
| STMN3 | -1.109467314 | 0.00258575 |
| WSCD2 | -1.109142487 | 0.03487531 |
| L1CAM | -1.104720873 | 0.03316446 |
| GNB5 | -1.104561249 | 0.01578567 |
| SYP | -1.093253531 | 0.01575962 |
| SNX10 | -1.085800487 | 0.02457063 |
| SGCZ | -1.082512887 | 0.01912745 |
| RAB6B | 1.075544117 | 0.01156651 |
| CASD1 | 1.088638639 | 0.03949756 |
| DLG3 | 1.090928855 | 0.0119178 |
| KIAA0319 | 1.091547208 | 0.01623589 |
| ST8SIA3 | 1.094862523 | 0.03119394 |
| USP25 | 1.095571643 | 0.01723263 |
| DBNDD1 | 1.09595027 | 0.04825815 |
| SIDT1 | 1.096103626 | 0.03107322 |
| TMEM181 | 1.096256626 | 0.04499498 |
| SYT5 | 1.101005394 | 0.03064953 |
| VAMP2 | 1.113451681 | 0.02142146 |
| CTXN1 | 1.113847116 | 0.03610438 |
| MYBPH | 1.118559179 | 0.0230048 |
| TMEM255B | 1.123084869 | 0.04112247 |
| CHST8 | 1.129277326 | 0.02310833 |
| C12orf56 | 1.137079352 | 0.00749243 |
| DOK6 | 1.142733638 | 0.04741384 |
| FLJ33534 | 1.146594587 | 0.01706151 |
| ARNT2 | 1.149681303 | 0.02738094 |
| GNRH2 | 1.152331295 | 0.0199295 |
| SCT | 1.157700647 | 0.04027366 |
| LIPC | 1.163955843 | 0.0477814 |
| SYCE1 | 1.167875417 | 0.01295983 |
| CCSER1 | 1.177778848 | 0.02080552 |
| GDA | 1.180071254 | 0.04531782 |
| MYO16 | 1.182572654 | 0.01860216 |
| STAP1 | 1.18698003 | 0.01123787 |
| DRD1 | 1.188251255 | 0.00402107 |
| PPM1M | 1.188792696 | 0.00584072 |
| GHR | 1.199225518 | 0.03084923 |
| ARNTL2 | 1.200145584 | 0.03602516 |
| NCAPG2 | 1.203491511 | 0.00048651 |
| HTR2A | 1.206111448 | 0.02016706 |
| OCM2 | 1.252407945 | 9.45E-06 |
| IL12RB2 | 1.26711447 | 0.04062216 |
| BMP6 | 1.272185801 | 0.00242816 |
| KL | 1.273417622 | 0.0124772 |
| LOC100506388 | 1.288300595 | 0.00068333 |
| LINC01140 | 1.37465835 | 0.00048586 |
| MET | 1.398560643 | 0.03061754 |
| ADRA1D | 1.427329962 | 3.56E-08 |
| CLIC6 | 1.438297493 | 0.01759967 |
| SASS6 | 1.459322874 | 2.45E-08 |
| FNDC1 | 1.459505439 | 0.02118734 |
| LOC100506731 | 1.550050146 | 8.01E-09 |
| ***PFC*** | | |
| MECOM | -1.426651881 | 3.96E-05 |
| GTF2I | -1.416507387 | 0.00032732 |
| PCDHB2 | -1.353562107 | 0.00022007 |
| ASPH | -1.346718819 | 0.0058077 |
| IKZF3 | -1.317912397 | 2.73E-05 |
| TPM2 | -1.298568322 | 0.00735598 |
| ANGPT4 | -1.28598657 | 0.00025008 |
| CYB5R3 | -1.25300981 | 2.73E-05 |
| LOC102724323 | -1.241561043 | 0.00136146 |
| ADAMTS19 | -1.24017991 | 0.00054542 |
| DHRS7C | -1.238641388 | 0.00054542 |
| ADAM28 | -1.227180055 | 0.00117565 |
| PIK3AP1 | -1.226375833 | 0.0225831 |
| FLI1 | -1.216317644 | 0.03657265 |
| SLC12A1 | -1.214676988 | 0.00115846 |
| IFNW1 | -1.194083229 | 0.00162802 |
| RPGRIP1L | -1.193418062 | 0.00392494 |
| POU2F2 | -1.190507046 | 0.00065326 |
| FLVCR2 | -1.189990826 | 0.0083991 |
| SLC26A8 | -1.189690274 | 0.00219903 |
| SLC8A2 | -1.181653508 | 0.00022019 |
| NPY6R | -1.168204365 | 0.00881761 |
| BUB1B | -1.168104367 | 0.0083991 |
| MICAL1 | -1.163651576 | 0.00156481 |
| THADA | -1.158531629 | 0.00308326 |
| JUN | -1.14853949 | 0.04632326 |
| RARB | -1.147842038 | 0.01191032 |
| SH2D1A | -1.142612997 | 0.00688304 |
| SYNPO | -1.11777717 | 0.00825522 |
| LOC101928658 | -1.116549162 | 0.01591172 |
| EPHB2 | -1.109215902 | 0.03494781 |
| CORO6 | -1.107840977 | 0.0199921 |
| MAP4K3 | 1.114169872 | 0.0225831 |
| TAF1D | 1.124250121 | 0.02236479 |
| MAP4K2 | 1.142188769 | 0.01591172 |
| SLITRK1 | 1.142244124 | 0.00187712 |
| TMEM52 | 1.153564384 | 0.03326161 |
| PP7080 | 1.154824838 | 0.01125922 |
| DDN | 1.157140114 | 0.00208234 |
| GBF1 | 1.157459342 | 0.00825522 |
| DUSP9 | 1.164872273 | 0.01929605 |
| EMX1 | 1.165952011 | 0.00090123 |
| MXRA5 | 1.18128607 | 0.01591172 |
| CBLN1 | 1.192582924 | 0.0074504 |
| PTPN13 | 1.194235719 | 0.03496685 |
| PPP1R9B | 1.196221684 | 0.00088732 |
| UNC5B-AS1 | 1.221198367 | 9.78E-05 |
| AOC3 | 1.224893657 | 0.00981603 |
| SYNDIG1L | 1.228263946 | 0.00056771 |
| LMNB1 | 1.23604535 | 0.00096876 |
| BMP6 | 1.311979719 | 2.73E-05 |
| MIDN | 1.319652716 | 0.00448504 |
| SCN5A | 1.350674347 | 2.11E-08 |
| CA5A | 1.367166871 | 2.45E-08 |
| MAGEE2 | 1.369735212 | 0.00022007 |
| TRPM4 | 1.426249931 | 5.06E-09 |
| KANK3 | 1.490847017 | 2.51E-08 |
| PAX7 | 1.848833662 | 1.04E-09 |
