# Supplementary figures and images for: Integrating Clinical and Genomic Analyses of Hippocampal-Prefrontal Circuit Disorder in Depression
Source: Front Genet. 2021 Feb 5;11:565749. doi: 10.3389/fgene.2020.565749 (PMC7893101; doi:10.3389/fgene.2020.565749)

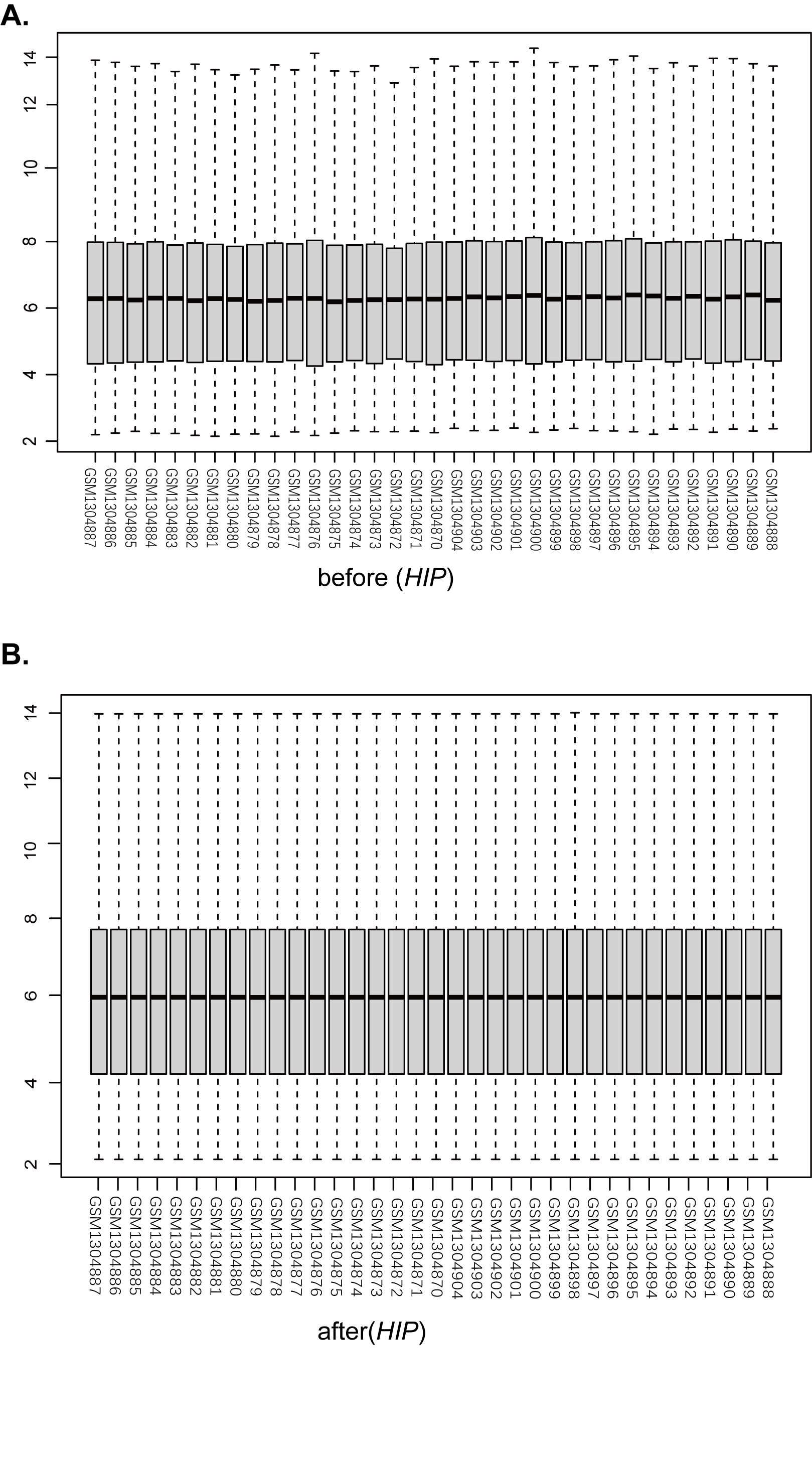

Supplement: Supplementary file 2 [file Image_1.TIF]

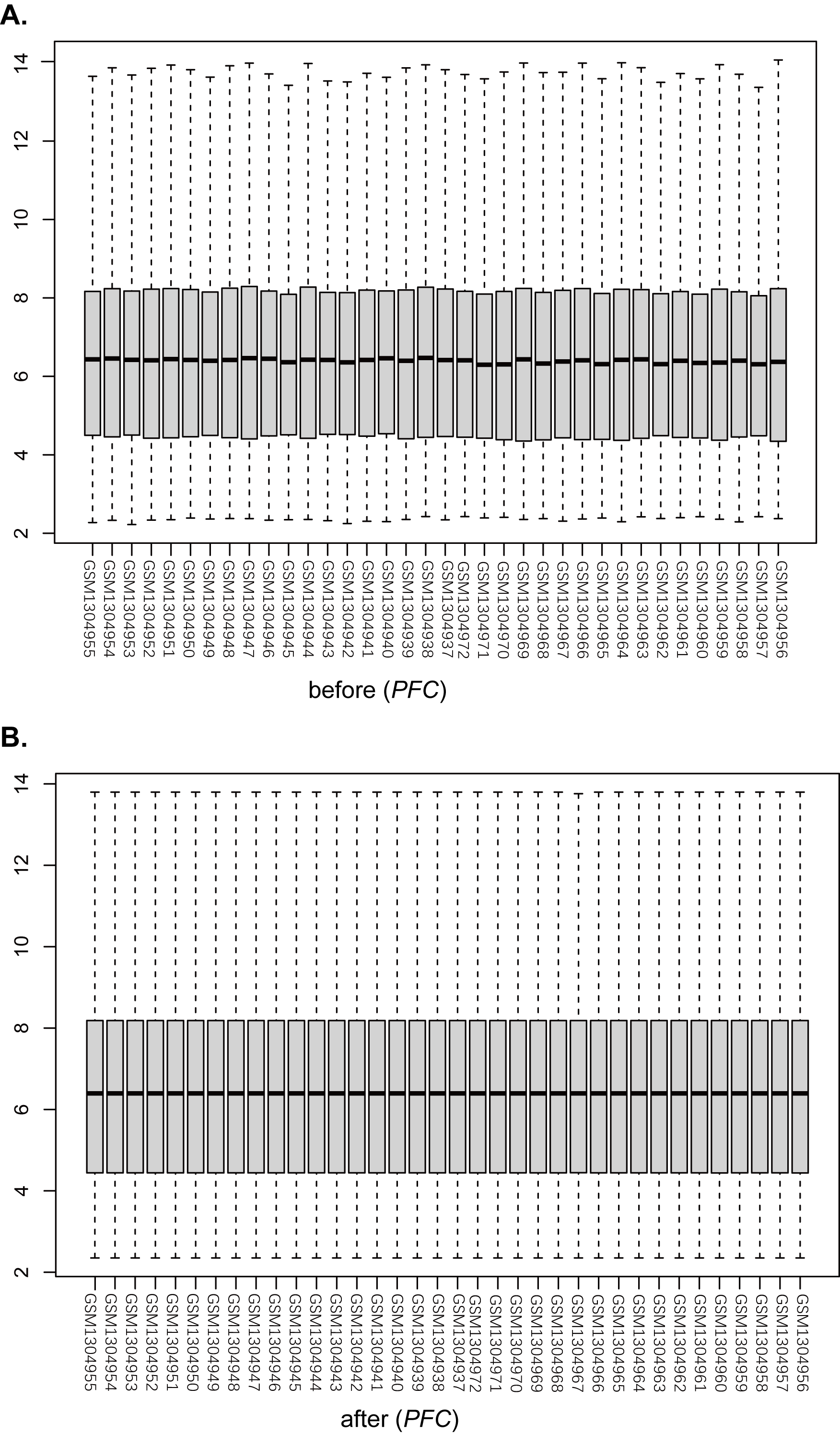

Supplement: Supplementary file 3 [file Image_2.TIF]
